# Cholest-5-ene- and Stigmasta-5,22-diene-Based 1,3-Oxathiolan-5-one Lactones and an Aminothiazole–Diosgenin Hybrid: Synthesis and Preliminary Antimicrobial Activity

**DOI:** 10.3390/molecules31132301

**Published:** 2026-07-01

**Authors:** Ahmad S. Barham, Khaled Q. Shawakfeh, Ali Elrashidi, Sameer Y. Jaradat

**Affiliations:** 1 Department of Chemistry, School of Science, The University of Jordan, Amman 11942, Jordan; 2 Department of Chemistry, Jordan University of Science and Technology, P.O. Box 3030, Irbid 22110, Jordan; 3 Electrical Engineering Department, College of Engineering, University of Business and Technology, Jeddah 23435, Saudi Arabia; a.elrashidi@ubt.edu.sa; 4 Engineering Mathematics Department, Faculty of Engineering, Alexandria University, Alexandria 21544, Egypt; 5 Basic Sciences Department, Imam Abdulrahman Bin Faisal University, Dammam 31441, Saudi Arabia; syjaradat@iau.edu.sa

**Keywords:** steroidal heterocycles, 1,3-oxathiolan-5-one, oxathiolanone lactone, aminothiazole, diosgenin, cholestane, stigmastane, S-alkylation, disc diffusion, antimicrobial

## Abstract

Novel steroidal heterocyclic derivatives were prepared from cholesterol, stigmasterol, and diosgenin scaffolds via concise, reagent-efficient synthetic routes. Two 1,3-oxathiolan-5-one derivatives bearing cholest-5-ene (**7**) and stigmasta-5,22-diene (**8**) cores were obtained from the corresponding steroidal ketones through hydrazone formation, phenyl isothiocyanate addition, and S-selective cyclization with chloroacetic acid in refluxing toluene. An aminothiazole–diosgenin hybrid (**12**) was independently prepared via regioselective α-bromination of an oxidized diosgenin intermediate followed by condensation with thiourea in ethanolic sodium ethoxide. The newly synthesized target compounds were characterized by FT-IR, ^1^H- and ^13^C-NMR spectroscopy, ESI mass spectrometry, and elemental (CHNS) analysis. The disc-diffusion assay against *S. aureus* and *E. coli* was retained only as a preliminary qualitative screen because no positive antibiotic control or MIC determination was included. Within this limited screen, compound **12** produced measurable 8.5–9.0 mm inhibition zones against both strains, supporting their prioritization for future standardized antimicrobial testing rather than establishing comparative potency.

## 1. Introduction

Steroids are structurally privileged scaffolds in medicinal chemistry, owing to their rigid polycyclic framework, metabolic stability, and inherent affinity for biological membranes and receptors [1,2]. The incorporation of heterocyclic motifs into steroidal frameworks has emerged as a productive strategy in drug design, since roughly two-thirds of approved small-molecule drugs contain heterocyclic rings [3,4,5]. Fusing nitrogen-, oxygen-, or sulfur-containing heterocycles to the A-ring of the steroid core has been shown to substantially modulate biological activity, yielding derivatives with antimicrobial, anti-inflammatory, antitumor, and enzyme-inhibitory profiles [6,7,8].

Among the steroidal scaffolds of synthetic interest, cholesterol, stigmasterol, and diosgenin are particularly attractive starting materials: they are inexpensive, commercially available in bulk, and carry well-defined stereochemistry that can be exploited in asymmetric synthesis. Stigmasterol, a plant phytosterol with documented cholesterol-lowering effects, and diosgenin, a spirostan-type sapogenin serving as the industrial precursor to numerous steroidal drugs, have been extensively derivatized [9,10,11,12,13,14]. Spirostan-type scaffolds have attracted particular attention as precursors to potent anticancer molecules, including the saponin OSW-1, and bis-steroidal natural products such as cephalostatins and ritterazines exhibit exceptional cytotoxicity toward human tumor cell lines [15].

Sulfur-containing heterocycles, in particular thiazoles, oxathiolanes, and related S/O-fused rings, represent a pharmacologically important class of compounds with well-documented antimicrobial activity [13,14]. The thiazole ring is present in several clinically used antibiotics, and its incorporation into steroidal frameworks has been reported to confer enhanced activity against both Gram-positive and Gram-negative bacteria [13,14]. Similarly, 1,3-oxathiolan-5-one (oxathiolanone) lactones are relatively underexplored in the steroidal context, and their synthesis from readily available steroid precursors has not been systematically reported.

In continuation of our interest in steroidal and related heterocyclic chemistry, we report here the synthesis of two novel 1,3-oxathiolan-5-one lactone derivatives bearing cholestane (**7**) and stigmastane (**8**) cores, prepared via an S-selective cyclization of steroidal carbothioamide intermediates with chloroacetic acid. In addition, we report the synthesis of an aminothiazole–diosgenin hybrid (**12**) via condensation of an α-bromoketone derived from diosgenin with thiourea. The key synthetic novelty lies in the S-alkylation/lactonization pathway leading to compounds **7** and **8**, which represents a previously unreported route to oxathiolanone-appended steroidal frameworks. All new compounds were characterized spectroscopically and evaluated in a preliminary disc-diffusion antimicrobial screen against S. aureus and E. coli; the biological data are interpreted only as exploratory because no positive antibiotic control or MIC values were obtained.

## 2. Results and Discussion

### 2.1. Synthesis of Hydrazone Derivatives ***3*** and ***4***

**Scheme 1:**
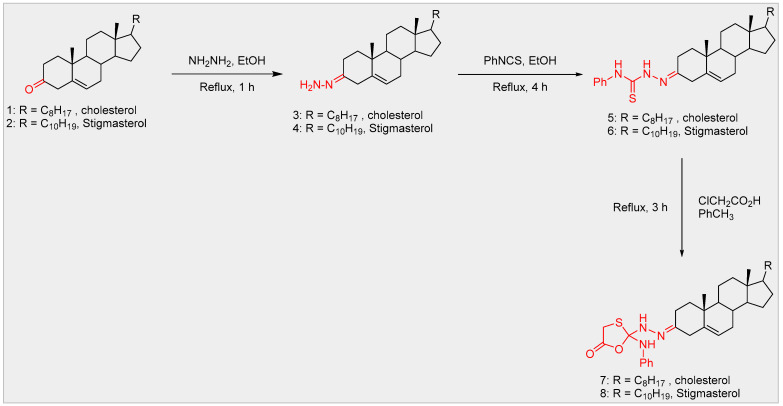
Synthetic route to hydrazone derivatives **3**,**4**, thiocarbamoyl adducts **5**,**6**, and cyclized 1,3-oxathiolan-5-one (lactone) products **7**,**8**.

Cholest-5-en-3-one (**1**) and stigmast-5,22-dien-3-one (**2**) were prepared by PCC oxidation of cholesterol and stigmasterol, respectively, following literature procedures [16]. Treatment of **1** and **2** with hydrazine hydrate in ethanol in the presence of triethylamine afforded the corresponding hydrazone derivatives **3** and **4**, in line with related steroidal hydrazone chemistry [17]. Good yields were obtained (75% and 70%, respectively) after column chromatography (Scheme 1). Reaction progress was monitored by TLC (EtOAc/hexane, 10:90, *v*/*v*).

Compound **3** was characterized by a D_2_O-exchangeable NH_2_ singlet at δ 5.95 ppm and a broad singlet at δ 5.33 ppm for the olefinic C-6 proton; the ^13^C-NMR spectrum confirmed hydrazone formation through the imine carbon signal at δ 155.1 ppm. Compound **4** showed the same key features, with the NH_2_ signal shifted to δ 5.84 ppm and an additional multiplet at δ 5.16–4.99 ppm from the C-22 and C-23 olefinic protons of the stigmastane side chain. Elemental analysis for both compounds agreed with the calculated values.

### 2.2. Synthesis of Carbothioamide Intermediates ***5*** and ***6***

Hydrazones **3** and **4** were reacted with phenyl isothiocyanate (1.0 equiv.) in refluxing ethanol to afford the acyclic carbothioamide adducts **5** and **6** in 32% and 61% yield, respectively. The ^1^H-NMR spectrum of **5** showed two D_2_O-exchangeable N–H signals at δ 9.33 (d, ^1^H, N–H adjacent to the C=N group) and δ 8.87–8.92 (m, ^1^H, N–H adjacent to the phenyl group), with ^13^C-NMR signals at δ 175.0 (C=S), 157.4 (C=N), and 124.1–129.1 ppm (aromatic carbons). The spectrum of **6** showed the same N–H pattern, together with the expected C-22/C-23 olefinic proton multiplet at δ 5.16−4.99 ppm and additional ^13^C signals at δ 138.0 (C-22) and 129.7 (C-23) ppm, confirming retention of the stigmastane Δ^22,23^ double bond. Elemental analysis for both compounds agreed within 0.4% nitrogen of the calculated values.

### 2.3. Synthesis of 1,3-Oxathiolan-5-one Lactones ***7*** and ***8***

**Scheme 2:**
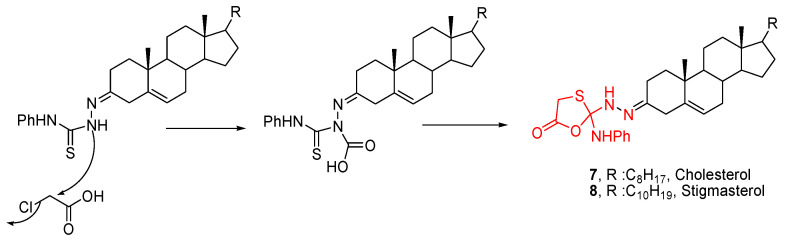
Proposed mechanism for the S-carboxymethylation/cyclization step of intermediates **5** and **6** with chloroacetic acid to give 1,3-oxathiolan-5-one (lactone) products **7** and **8**.

Intermediates **5** and **6** were cyclized with chloroacetic acid in refluxing toluene to investigate fused heterocycle formation on the steroid A-ring. Two pathways were possible: N-alkylation of the terminal N–H to give a thiazolidin-4-one ring, or S-alkylation of the thioamide sulfur followed by intramolecular lactonization to give a 1,3-oxathiolan-5-one (Scheme 2). The latter pathway is chemically reasonable because the softer thioamide sulfur is expected to react preferentially with the electrophilic methylene carbon of chloroacetic acid, followed by intramolecular O-acylation to give lactones **7** and **8** in 50% and 54% yield, respectively. Close steroidal precedents for this lactone-forming transformation appear limited, and compounds **7** and **8** were sufficiently stable for isolation, purification, and acquisition of FT-IR, NMR, ESI-MS, and elemental-analysis data under the reported laboratory conditions.

The 1,3-oxathiolan-5-one assignment, rather than the thiazolidin-4-one regioisomer, is supported by the available orthogonal spectral and analytical data. First, the FT-IR C=O stretching absorptions at 1728 cm^−1^ (**7**) and 1722 cm^−1^ (**8**) are consistent with lactone carbonyl absorption. Second, the ^13^C-NMR lactone carbonyl resonances at δ 177.9 (**7**) and 172.9 ppm (**8**) support the formation of an ester-type carbonyl environment. Third, both compounds retain two D_2_O-exchangeable N–H protons (**7**: δ 8.24 and 4.24 ppm; **8**: δ 7.34 and 4.10 ppm), which is consistent with S- rather than N-participation in ring closure. Fourth, the –SCH_2_CO– methylene singlet at δ 4.12–4.14 ppm (2H), together with ESI-MS molecular-ion peaks (**7**: *m*/*z* 592 [M + H]^+^; **8**: *m*/*z* 618 [M + H]^+^) and satisfactory CHNS analyses, supports the proposed structures. Unfortunately, 2D NMR experiments could not be performed because the appropriate probe was unavailable.

### 2.4. Synthesis of Aminothiazole–Diosgenin Hybrid ***12***

**Scheme 3:**
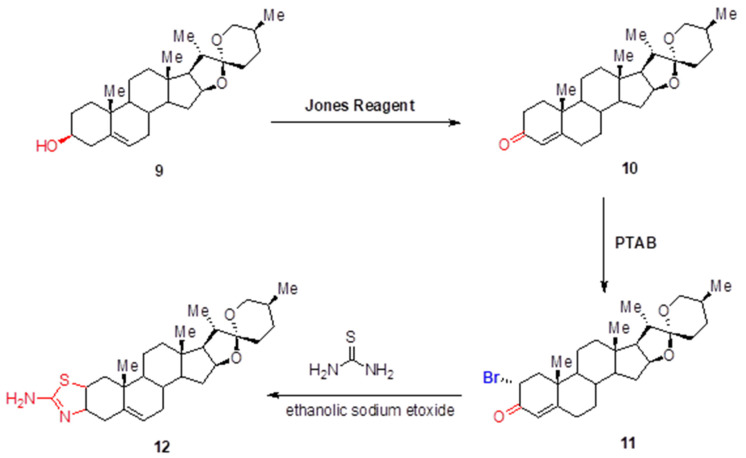
Synthesis of aminothiazole-diosgenin **12**.

Thiazole-containing heterocycles have attracted steady interest in medicinal chemistry for their antimicrobial and antitumor activities. To complement the oxathiolanone series, an aminothiazole–diosgenin hybrid was prepared by an independent route. Therefore, treatment of ketone **10** with phenyltrimethylammonium tribromide (PTAB) resulted in selective bromination, yielding α-bromo ketone **11** as the thermodynamic product. The spectral data obtained for **11** were consistent with those reported previously in the literature [12,18]. Condensation of **11** with thiourea in ethanolic sodium ethoxide under reflux then gave the target aminothiazole–diosgenin hybrid **12** (Scheme 3) [13,14].

The ^1^H-NMR spectrum of **12** (DMSO-*d*_6_) showed a D_2_O-exchangeable NH_2_ singlet at δ 5.92 ppm and a doublet at δ 5.37 ppm (*J* = 5.42 Hz) for the C-6 olefinic proton. The ^13^C-NMR spectrum displayed the aminothiazole C-2 carbon (S–C=N) at δ 163.3 ppm along with characteristic diosgenin spiroketal signals at δ 107.0 (C-22) and 68.9 ppm (C-26). ESI-MS (*m*/*z* 471 [M + H]^+^) and elemental analysis confirmed the structure.

### 2.5. Preliminary Antimicrobial Activity

Compounds **7**, **8**, and **12** were evaluated in a preliminary qualitative disc-diffusion assay against *S. aureus* ATCC 25923 and *E. coli* ATCC 25922 to obtain an initial indication of their biological relevance (Table 1). DMSO was used as the negative control at 6.3 μL/disc. After incubation, inhibition zones were measured in millimeters. No positive antibiotic control was included, and MIC values were not determined; therefore, the assay was used only as an exploratory screen and cannot validate assay sensitivity, establish potency, or support direct comparison with standard antimicrobial agents. The modest difference between compounds **7** and **8** may reflect the influence of the steroidal core, including the C-22/C-23 side-chain unsaturation in **8**, although systematic dose–response studies are required before any structure–activity relationship can be established. Within this limited qualitative screen, compounds **7** and **12** gave the largest inhibition zones and may therefore be prioritized for MIC determination, positive-control benchmarking, and broader antimicrobial evaluation.

## 3. Materials and Methods

### 3.1. Materials and Physical Measurements

All reagents and chemicals were purchased from commercial suppliers and used without further purification unless noted otherwise. Solvents were dried and distilled using standard drying agents and procedures. Melting points (mp) were measured using an electrothermal digital (IA9000) apparatus (Rochford, Essex, UK). FT-IR spectra were conducted on a JASCO FT-IR (Tokyo, Japan) spectrophotometer using KBr discs or CHCl_3_ solutions as indicated. ^1^H- and ^13^C-NMR analyses were conducted on a 400 MHz Bruker AVANCE spectrometer (Rheinstetten, Germany). Chemical shifts (δ) were given in ppm with tetramethylsilane (TMS) used as the internal reference. Mass spectrometric measurements (MS) were conducted using an LC-MSD spectrometer (Trap-00125) equipped with an ion source (ESI type) (Agilent Technologies, Santa Clara, CA, USA). Monitoring the reaction progress was achieved by thin-layer chromatography (TLC) prepared on silica gel plates of 60 F254. Elemental analyses (C, H, N, S) were conducted using a EuroEA Elemental Analyzer (Milan, Italy). *S. aureus* (ATCC 25923) and *E. coli* (ATCC 25922) were obtained from the American Type Culture Collection (ATCC, Manassas, VA, USA).

### 3.2. General Procedure for the Preparation of Hydrazone Derivatives ***3*** and ***4***

Hydrazine hydrate (3.0 mL) and triethylamine (NEt_3_, 1.0 mL) were added to a solution of 1.0 mmol ketone **1** or **2** in EtOH (96%, 5 mL). The mixture was stirred under reflux for 1 h. After cooling to ambient temperature, the solvent was evaporated under vacuum, giving a yellow precipitate. The crude product was taken up in absolute EtOH and filtered. The filtrate was concentrated under reduced pressure to give a yellow solid, which was washed with water and purified by column chromatography to afford products **3** or **4**.

### 3.3. General Procedure for the Reaction of Hydrazones with Phenyl Isothiocyanate (***5*** and ***6***)

In 30 mL of ethanol, a mixture of compounds **3** or **4** (0.005 mol) and phenyl isothiocyanate (0.67 g, 0.005 mol) was refluxed for 4 h. After completion, the solvent was removed under reduced pressure. The resulting semisolid residue was recrystallized from EtOH and then refined by flash chromatography to give compounds **5** and **6** as pale-yellow powders.

### 3.4. General Procedure for the Cyclization of ***5*** and ***6*** with Chloroacetic Acid to Afford ***7*** and ***8*** (1,3-Oxathiolan-5-one Lactones)

A toluene solution of compound **5** or **6** (0.375 mmol, 10 mL) was combined with a solution of chloroacetic acid (0.749 mmol) in dry toluene (10 mL). The mixture was refluxed for 3 h, then concentrated under vacuum. The resulting solid was isolated and recrystallized from ethanol to give compounds **7** and **8**.

### 3.5. Synthesis of Intermediates ***10*** and ***11*** and Target Aminothiazolyl Derivative ***12***

At 10 °C, compound **10** was obtained from compound **9** (1.0 g, 2.41 mmol) via Jones oxidation in acetone (50 mL). The reaction was maintained below 20 °C and stirred for 30 min, then washed with brine and extracted with CHCl_3_ (3 × 25 mL). The combined organic extracts were dried over anhydrous Na_2_SO_4_ and concentrated under reduced pressure. Purification of the crude product by column chromatography (20% EtOAc/hexane) provided compound **10** as a white solid (Yield: 0.95 g, 95%); mp 193–194 °C (lit. mp 194–196 °C [10]).

Compound **11** was prepared by adding a solution of phenyltrimethylammonium tribromide (PTAB, 0.23 g, 0.63 mmol, 1.3 equiv.) in THF (7 mL) rapidly to a THF solution (20 mL) of compound **10** (0.20 g, 0.48 mmol) at 20 °C. An orange mixture was obtained, which quickly produced a heavy precipitate and changed to a beige-colored suspension within approximately 6 min. The reaction was then quenched with 10 mL of brine and extracted with CHCl_3_ (2 × 20 mL). The combined organic phases were dried over anhydrous Na_2_SO_4_, concentrated under reduced pressure, and purified by column chromatography (15% EtOAc/hexane) to afford compound **11** as a white solid; mp 159–161 °C (lit. mp 160–163 °C [12,18]).

To prepare compound **12**, compound **11** (0.34 g, 0.69 mmol) was added to a freshly prepared sodium ethoxide solution (0.25 mol L^−1^, prepared by dissolving sodium metal (0.23 g, 10 mmol) in absolute ethanol (40 mL)) [13], followed by thiourea (0.7 g, 9.0 mmol). The reaction mixture was refluxed for approximately 24 h and then concentrated under reduced pressure. The crude material was purified by recrystallization from ethanol followed by column chromatography using 20% EtOAc/hexane, yielding compound **12** as a pale-yellow solid (0.27 g, 57%).

### 3.6. Characterization Data

Compound **3**: 0.31 g (75%), mp 245–247 °C. FT-IR (KBr, cm^−1^): 3297, 3057, 2943, 2908, 2868, 2852, 1655, 1606, 1542, 1497, 1256. ^1^H-NMR (400 MHz, CDCl_3_) δ (ppm): 5.95 (s, 2H, NH_2_, D_2_O exchangeable), 5.33 (br s, 1H, C-6), 2.84 (dd, 2H, C-4, J_HH_ = 16.4, 2.4 Hz), 2.53 (m, 1H, C-2β-H), 2.26 (m, 1H, C-2α-H), 1.98 (s, 3H, C-21), 1.20 (s, 3H, C-19), 0.71 (s, 3H, C-18). ^13^C-NMR (CDCl_3_) δ (ppm): 155.1 (C-3, C=N), 144.1 (C-5), 125.2 (C-6), 58.5 (C-17), 56.0 (C-14), 48.8 (C-9), 40.9 (C-13), 36.8 (C-20), 36.4 (C-10), 36.1 (C-1), 36.1 (C-12), 35.4 (C-24), 35.2 (C-4), 34.1 (C-23), 33.8 (C-7), 33.1 (C-22), 31.1 (C-25), 30.9 (C-8), 27.9 (C-16), 25.4 (C-2), 25.1 (C-15), 21.0 (C-21), 21.0 (C-11), 20.7 (C-19), 20.0 (C-26), 16.9 (C-27), 12.1 (C-18). Anal. Calc. for C_27_H_46_N_2_ (398.67): C, 81.34%; H, 11.63%; N, 7.03%. Found: C, 81.14%; H, 11.58%; N, 7.34%.

Compound **4**: 0.30 g (70%), mp 248−249 °C. FT-IR (KBr, cm^−1^): 3277, 3067, 2953, 2928, 2892, 2808, 1625, 1616, 1552, 1477, 1266. ^1^H-NMR (400 MHz, CDCl_3_) δ (ppm): 5.84 (s, 2H, NH_2_, D_2_O exchangeable), 5.37 (br s, 1H, C-6), 5.16−4.99 (m, 2H, C-22, 23), 2.79 (dd, 2H, C-4, J_HH_ = 16.4, 2.4 Hz), 2.43 (m, 1H, C-2β-H), 2.21 (m, 1H, C-2α-H), 2.02 (s, 3H, C-21), 1.33 (s, 3H, C-19), 0.73 (s, 3H, C-18). ^13^C-NMR (CDCl_3_) δ (ppm): 157.4 (C-3, C=N), 147.1 (C-5), 137.1 (C-22), 129.1 (C-23), 125.2 (C-6), 56.0 (C-14), 55.5 (C-17), 54.4 (C-24), 46.8 (C-9), 42.8 (C-20), 40.9 (C-13), 36.4 (C-10), 36.1 (C-1), 36.1 (C-12), 35.2 (C-4), 33.8 (C-7), 31.1 (C-25), 30.9 (C-8), 27.9 (C-16), 27.1 (C-28), 25.4 (C-2), 25.1 (C-15), 21.3 (C-21), 21.0 (C-11), 21.0 (C-26), 20.7 (C-19), 18.9 (C-27), 12.5 (C-29), 12.1 (C-18). Anal. Calc. for C_29_H_48_N_2_ (424.7): C, 82.01%; H, 11.39%; N, 6.60%. Found: C, 81.84%; H, 11.38%; N, 6.80%.

Compound **5**: 0.42 g (32%). mp 148–149 °C. FT-IR (CHCl_3_, cm^−1^): 2987, 2870, 1720. ^1^H-NMR (400 MHz, CDCl_3_) δ (ppm): 9.33 (d, 1H, NH, D_2_O exchangeable), 8.87−8.92 (m, 1H, NH, D_2_O exchangeable), 7.21–7.64 (m, 5H, aromatic protons), 5.84 (br s, 1H, C-6), 2.63 (br s, 2H, C-4), 2.33 (m, 1H, C-2β-H), 2.27 (m, 1H, C-2α-H), 1.82 (s, 3H, C-21), 1.25 (s, 3H, C-19), 0.85 (s, 3H, C-18). ^13^C-NMR (CDCl_3_) δ (ppm): 175.0 (C=S), 157.4 (C-3, C=N), 147.7 (C-5), 124.1–129.1 (aromatic carbons), 123.1 (C-6), 57.8 (C-17), 56.2 (C-14), 49.4 (C-9), 40.4 (C-13), 39.1 (C-1), 37.5 (C-12), 36.3 (C-20), 35.9 (C-10), 35.1 (C-24), 34.4 (C-4), 34.7 (C-23), 33.1 (C-7), 33.0 (C-22), 31.8 (C-25), 31.8 (C-8), 28.8 (C-16), 25.3 (C-15), 25.2 (C-2), 21.1 (C-11), 21.0 (C-21), 21.0 (C-26), 19.9 (C-19), 16.7 (C-27), 12.1 (C-18). Anal. Calc. for C_34_H_51_N_3_S (533.86): C, 76.49%; H, 9.63%; N, 7.87%; S, 6.01%. Found: C, 76.19%; H, 9.68%; N, 7.94%; S, 6.14%.

Compound **6**: 0.40 g (61%). mp 150–152 °C. FT-IR (CHCl_3_, cm^−1^): 3192, 2957, 2870, 1597, 1514. ^1^H-NMR (400 MHz, CDCl_3_) δ (ppm): 9.33 (d, 1H, NH, D_2_O exchangeable), 8.87–8.92 (m, 1H, NH, D_2_O exchangeable), 7.21–7.64 (m, 5H, aromatic protons), 5.84 (br s, 1H, C-6), 5.16–4.99 (m, 2H, C-22 and C-23), 2.63 (br s, 2H, C-4), 2.33 (m, 1H, C-2β-H), 2.27 (m, 1H, C-2α-H), 1.82 (s, 3H, C-21), 1.25 (s, 3H, C-19), 0.85 (s, 3H, C-18). ^13^C-NMR (CDCl_3_) δ (ppm): 175.0 (C=S), 157.4 (C-3, C=N), 147.7 (C-5), 138.0 (C-22), 129.7 (C-23), 124.1 (C-6, overlapping with aromatic carbons), 124.1–129.1 (aromatic carbons), 56.2 (C-14), 55.8 (C-17), 54.1 (C-24), 46.4 (C-9), 42.3 (C-20), 40.4 (C-13), 35.9 (C-10), 37.5 (C-12), 36.3 (C-1), 32.4 (C-4), 33.1 (C-7), 31.8 (C-25), 31.8 (C-8), 28.8 (C-16), 27.0 (C-28), 24.2 (C-2), 25.3 (C-15), 21.0 (C-21), 21.1 (C-11), 21.0 (C-26), 19.9 (C-19), 18.9 (C-27), 12.2 (C-29), 12.1 (C-18). Anal. Calc. for C_36_H_53_N_3_S (559.90): C, 77.23%; H, 9.54%; N, 7.51%; S, 5.73%. Found: C, 76.99%; H, 9.60%; N, 7.71%; S, 5.74%.

Compound **7**: 0.11 g (50%). Amorphous solid. FT-IR (KBr, cm^−1^): 3469, 3269, 3053, 2929, 2867, 1728, 1560. ^1^H-NMR (400 MHz, CDCl_3_) δ (ppm): 8.24 (br s, 1H, NH, D_2_O exchangeable), 7.15–7.55 (m, 5H, aromatic protons), 5.86 (m, 1H, C-6), 4.24 (br s, 1H, NH-Ph, D_2_O exchangeable), 4.12 (s, 2H, CH_2_-CO), 2.12 (m, 2H, C-4), 2.10 (m, 1H, C-2β-H), 2.06 (m, 1H, C-2α-H), 1.30 (s, 3H, C-21), 1.15 (s, 3H, C-19), 0.89 (s, 3H, C-18). ^13^C-NMR (CDCl_3_) δ (ppm): 177.9 (C=O, lactone), 165.3 (C-3), 148.3 (C-5), 135.0 (quaternary C), 126.0 (C-6, overlapping with aromatic carbons), 120.2–129.2 (aromatic carbons), 58.2 (C-14), 56.3 (C-17), 49.7 (C-9), 41.1 (C-13), 37.7 (C-1), 36.9 (C-20), 35.9 (C-10), 37.0 (C-12), 35.0 (C-24), 34.1 (C-23), 33.1 (C-4), 33.1 (C-7), 32.4 (C-22), 31.0 (C-25), 30.9 (C-8), 28.7 (C-16), 28.2 (CH_2_–S), 26.1 (C-2), 25.3 (C-15), 21.0 (C-21), 21.1 (C-11), 21.2 (C-26), 22.5 (C-19), 16.7 (C-27), 12.1 (C-18). ESI-MS (*m*/*z*): 592 [M + H]^+^. Anal. Calc. for C_36_H_53_N_3_O_2_S (591.89): C, 73.05%; H, 9.03%; N, 7.10%; S, 5.42%. Found: C, 73.14%; H, 8.98%; N, 7.54%; S, 5.44%.

Compound **8**: 0.12 g (54%). Amorphous solid. FT-IR (KBr, cm^−1^): 3426, 3269, 3016, 2922, 2867, 1722, 1560. ^1^H-NMR (400 MHz, CDCl_3_) δ (ppm): 7.34 (br s, 1H, NH, D_2_O exchangeable), 7.15–7.26 (m, 5H, aromatic protons), 5.91 (m, 1H, C-6), 4.10 (br s, 1H, NH-Ph, D_2_O exchangeable), 4.14 (s, 2H, CH_2_-CO), 2.12 (m, 2H, C-4), 2.10 (m, 1H, C-2β-H), 2.06 (m, 1H, C-2α-H), 1.81 (s, 3H, C-21), 1.25 (s, 3H, C-19), 0.85 (s, 3H, C-18). ^13^C-NMR (CDCl_3_) δ (ppm): 172.9 (C=O), 159.3 (C-3), 148.3 (C-5), 137.4 (C-22), 135.0 (quaternary C), 129.2 (C-23, overlapping with aromatic carbons), 126.0 (C-6, overlapping with aromatic carbons), 120.2–129.2 (aromatic carbons), 58.4 (C-14), 56.3 (C-17), 53.6 (C-24), 45.7 (C-9), 41.9 (C-20), 41.4 (C-13), 35.0 (C-10), 37.4 (C-12), 35.7 (C-1), 33.1 (C-4), 33.4 (C-7), 31.0 (C-25), 31.8 (C-8), 29.1 (C-16), 27.0 (C-28), 27.1 (C-2), 25.1 (C-15), 21.0 (C-21), 21.1 (C-11), 21.2 (C-26), 22.5 (C-19), 18.9 (C-27), 12.5 (C-29), 12.1 (C-18). ESI-MS (*m*/*z*): 618 [M + H]^+^. Anal. Calc. for C_38_H_55_N_3_O_2_S (617.94): C, 73.86%; H, 8.97%; N, 6.80%; S, 5.19%. Found: C, 73.74%; H, 8.98%; N, 7.14%; S, 5.14%.

Compound **12**: 0.27 g (57%). Pale-yellow solid, mp 259–262 °C. FT-IR (KBr, cm^−1^): 3442, 3280−3340, 3053, 2929, 2867, 1632, 1598, 1564, 1524, 1505. ^1^H-NMR (400 MHz, DMSO-*d*_6_) δ: 5.92 (D_2_O exchangeable, s, 2H, –NH_2_), 5.37 (d, 1H, *J* = 5.42 Hz, 6-H), 2.76 (m, 1H, 2α-H), 2.21 (dd, 1H, *J* = 5.42 Hz, 3α-H), 1.12 (s, 3H, 18-Me), 0.79 (s, 3H, 19-Me). ^13^C-NMR (DMSO-*d*_6_) δ (ppm): 163.3 (S–C=N, C-2 of thiazole), 140.7 (C-5), 121.9 (C-4), 107.0 (spiroketal C-22), 81.1 (C-16), 68.9 (C-26), 63.0 (C-17), 61.1 (C-3), 54.0 (C-14), 52.9 (C-9), 44.1 (C-1), 41.9 (C-20), 39.7 (C-13), 39.8 (C-6), 38.8 (C-12), 33.9 (C-10), 33.0 (C-8), 32.0 (C-15), 31.5 (C-23), 31.2 (C-7), 31.0 (C-25), 28.8 (C-24), 28.4 (C-2), 22.0 (C-11), 17.0 (C-27), 16.5 (C-18), 15.1 (C-21), 12.3 (C-19). ESI-MS (*m*/*z*): 471 [M + H]^+^. Anal. Calc. for C_28_H_42_N_2_O_2_S (470.71): C, 71.45%; H, 8.99%; N, 5.95%; S, 6.81%. Found: C, 71.92%; H, 8.98%; N, 6.14%; S, 6.44%.

## 4. Conclusions

Novel steroidal heterocyclic derivatives were synthesized from cholesterol, stigmasterol, and diosgenin via concise, reagent-efficient routes. Two 1,3-oxathiolan-5-one lactone derivatives bearing cholestane (**7**) and stigmastane (**8**) cores were obtained through S-selective cyclization of carbothioamide intermediates with chloroacetic acid in refluxing toluene. An aminothiazole–diosgenin hybrid (**12**) was independently prepared via regioselective α-bromination of an oxidized diosgenin intermediate followed by condensation with thiourea. The newly synthesized target compounds were characterized by FT-IR, ^1^H- and ^13^C-NMR spectroscopy, ESI mass spectrometry, and elemental analysis. The preliminary disc-diffusion screen identified compounds **7** and **12** as producing the largest zones in the limited dataset; however, because the assay was conducted without a positive antibiotic control and without MIC determination, these results should be regarded as exploratory only. Future work will require standardized positive-control benchmarking, MIC determination, expanded bacterial panels, and additional 2D/heteronuclear NMR support for key structural assignments.

## Figures and Tables

**Table 1:** Zone of inhibition (mm) for selected compounds against *E. coli* and *S. aureus* by disc-diffusion screening; values are reported as mean ± SD from three independent measurements.

Microorganism	7 (mm)	8 (mm)	12 (mm)	Negative Control
*E. coli*	9.0 ± 0.0	8.0 ± 0.0	8.5 ± 0.0	No inhibition (DMSO)
*S. aureus*	9.0 ± 0.0	7.5 ± 0.0	9.0 ± 0.0	No inhibition (DMSO)

Values represent mean ± SD from three independent measurements. Identical recorded values across replicates gave SD = 0.0 mm. DMSO was used as the negative control (6.3 μL/disc). Because no positive antibiotic control or MIC determination was included, the results should be interpreted only as exploratory and should not be used for comparative potency claims.

## Data Availability

The data supporting the findings of this study are available from the corresponding authors upon reasonable request.
